# Classification Method for Viability Screening of Naturally Aged Watermelon Seeds Using FT-NIR Spectroscopy

**DOI:** 10.3390/s19051190

**Published:** 2019-03-08

**Authors:** Jannat Yasmin, Mohammed Raju Ahmed, Santosh Lohumi, Collins Wakholi, Moon S. Kim, Byoung-Kwan Cho

**Affiliations:** 1 Department of Biosystems Machinery Engineering, College of Agricultural and Life Science, Chungnam National University, 99 Daehak-ro, Yuseong-gu, Daejeon 34134, Korea; ahmed37.bau@hotmail.com (J.Y.); raju_115569@yahoo.com (M.R.A.); santosh.sanny123@gmail.com (S.L.); wcoln@yahoo.com (C.W.); 2 Environmental Microbial and Food Safety Laboratory, Agricultural Research Service, U.S. Department of Agriculture, Powder Mill Rd. Bldg. 303, BARC-East, Beltsville, MD 20705, USA; Moon.Kim@ars.usda.gov

**Keywords:** seed viability, watermelon, spectroscopic analysis, near infrared, nondestructive measurement

## Abstract

Viability analysis of stored seeds before sowing has a great importance as plant seeds lose their viability when they exposed to long term storage. In this study, the potential of Fourier transform near infrared spectroscopy (FT-NIR) was investigated to discriminate between viable and non-viable triploid watermelon seeds of three different varieties stored for four years (natural aging) in controlled conditions. Because of the thick seed-coat of triploid watermelon seeds, penetration depth of FT-NIR light source was first confirmed to ensure seed embryo spectra can be collected effectively. The collected spectral data were divided into viable and nonviable groups after the viability being confirmed by conducting a standard germination test. The obtained results showed that the developed partial least discriminant analysis (PLS-DA) model had high classification accuracy where the dataset was made after mixing three different varieties of watermelon seeds. Finally, developed model was evaluated with an external data set (collected at different time) of hundred samples selected randomly from three varieties. The results yield a good classification accuracy for both viable (87.7%) and nonviable seeds (82%), thus the developed model can be considered as a “general model” since it can be applied to three different varieties of seeds and data collected at different time.

## 1. Introduction

The seed can be determined as a small embryonic plant with stored food enclosed with a cover or seed coat. The progressive deterioration of the seed structure and function over time can be termed as the seed aging which leads to the ultimate death of the organism. People who are involved in crop production, need the first real opportunity for the practical evaluation of seed quality at time of sowing [1]. Hence, viability is an important consideration to ensure higher crop yield. Seed viability can be termed as the measurement of alive seeds which are able to develop into plants. During germination some seeds might not germinate due to dormancy or sleep period. Seed dormancy is a condition that prevents a seed being germinated even under optimal environmental conditions. According to Nikolaeva (1977) [2], to bring a seed out from a dormant state, we need to break two general type of dormancy factors (endogenous and exogenous). A seed which has deformed internal chemical or metabolic conditions along with an immature embryo that is not fully developed and prevents germination is in the chemical dormancy state, which is under exogenous dormancy. 

At the beginning of planting the seeds, the germination capacity of the seeds must be known to ensure the desired population of seedlings. Seed viability denotes the high quality, and physiological and genetic purity which are required to produce uniform seedling emergence. Seed viability analysis is a dynamic activity which is constantly evolving and is characterized by continuous improvements and standardization of the developing procedures.

Watermelon is a warm seasonal annual crop, therefore, an elevated temperature and high humidity may induce seed deterioration within the fruit [3]. Seedless watermelon seed (also called triploid watermelon) production is almost the same as seeded (diploid) watermelon. But the seed coat of triploid watermelon is thicker than diploid seeds, and in addition, production cost is higher due to the expensive seed [4]. Though seed viability is one of the most important factors for crop production, seed deterioration may be a reason for poor germination ability which will reduce the production rate of triploid watermelon. Due to being a living material, seeds need proper monitoring to assure the viability during storage [5] because seeds are the storehouse of chemicals to reserve food for the next generation plant. Previous researches revealed that seed viability is impacted by age because of the oxidation of polyunsaturated fatty acid that reduces the seed’s membrane permeability and fluidity which leads to change in enzymatic activity [6]. Different biochemical and physiological changes happen due to deterioration, which hampers the seed quality [7].

Seed aging and viability are affected by several factors through the seed production and storage procedure. A published research study revealed that physiological changes occur in the seed due to a long term storage period that affects the seed viability [8]. Seed viability tests are considered one of the primary tasks in crop production, and are widely used, as they give a good indication of potential harvesting. This is the reason for increasing the use of viability tests for seed-producing industries to determine the seed quality [9]. A viability test reduces risks and losses, which improves market competition. It is impossible to separate large volumes of nonviable seeds, either using human eyesight or low capability instruments [10]. The conventional methods for testing seed viability, the germination test and the tetrazolium test (TZ), are time consuming and destructive for the seed sample [11]. Sometimes the outer surface of a seed does not change but during the germination procedure, it cannot get germinated. Therefore, for business optimization, seed supplier companies need a fast seed viability testing and classification technique which can handle bulk volumes of seeds efficiently. Regarding this issue, it is of increasing interest to develop a method that can quickly and efficiently assess the seed quality before marketing. 

The quick indication of seed viability has many advantages for seed-producing industries that will allow quick decision making. Determination of seed quality is a very meaningful consideration for producers who need to be able to predict seed viability by an appropriate methodology in a precise fashion. Chemometric analysis combined with FT-NIR spectroscopic analysis is a renowned analytical approach to identify viable and non-viable seeds due to its simplicity, rapidity, and ease of sample preparation. 

The application of Fourier transform near infrared (FT-NIR) spectroscopy is increasing in the food sector due to being a chemical free, non-destructive, reliable, and fast technique which is based on the absorption of NIR light by organic compounds. Previous literature review reveals that FT-NIR is effective at discriminating normal seed in wheat [12], and at nondestructive classification of artificially aged *Pinus plata* seed [13], radish seed [14], and watermelon seed [15]. However, artificially aged seeds are not representative of naturally aged seeds. Naturally aged seeds provide a real scenario of seed response to the conditions to which they are exposed [16].

Several previous researches were performed on both artificially and naturally aging of the same seed and revealed that, for the artificially aged seed, significant declination occurs compared to seeds in conventional storage conditions. The amount of α-tocopherol (an antioxidant that blocks the entrance of oxygen into the internal tissue of seed which causes deterioration) was present in high amounts in artificially aged seeds compared to naturally aged seeds where the experiment was done on four types of soybean cultivars [17]. Petruzzelli and Carella [18] conducted a research in 1983 on artificial and natural aged wheat seed and proved that artificially aging of seed amplifies the deteriorative changes for seed than naturally aging. Another research study aimed to evaluate the association between artificial and natural aging of four cultivars of coffee seeds and proved that greater deterioration happens for artificial aging compared with natural aging [19]. Hence artificially aged seed give higher prediction accuracy. Therefore, this study used naturally aged triploid (seedless) watermelon seeds to evaluate the seed viability.

For the analysis of complex spectral data, multivariate analysis is a chemometric tool used to determine each component [20]. Partial least square (PLS) is the most commonly used method during multivariate calibration which is based on data reduction and inverse calibration. During the FT-NIR spectroscopic analysis, the spectra is used as a fingerprint to identify it, and measures the vibration in energy absorption that depends on the chemical composition and moisture content of the sample. 

In this context, the aim of this study is to develop a general model based on the spectral difference obtained from the FT-NIR spectroscopic technique combined with multivariate data analysis of naturally aged triploid watermelon seed varieties. Afterwards, this model was further used to detect naturally aged viable and nonviable seed from the absolute new data set. 

## 2. Materials and Methods

### 2.1. Seed Samples

**Figure 1:**
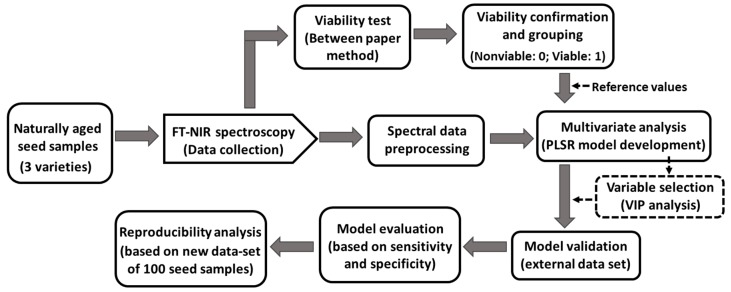
Procedure used to generate the generalized classification model using FT-NIR spectroscopic technique.

The three varieties of triploid watermelon seed sample; commercially termed as, 3X:BH4X415(3X), 3X:NP114013G4(4X), and 3X: SN3615 (4X) hereafter referred to as the V1, V2, and V3, respectively, were collected from Partner Seed Company (Gyeonggi-do, Korea). Due to being naturally aged, seeds of 2014 were used for making a model. The seed samples were stored in a small room particularly designed for seed lots under the management of Korea Seed and Variety Service (KSVS). Seeds were sealed in a plastic container and the room temperature was controlled around 5 °C. Six hundred seeds were used randomly from each variety (total 1800 seeds from 3 varieties). The working procedure to build the general model developed using FT-NIR spectroscopy based on their chemical compositional change related with viability, shows in Figure 1.

### 2.2. FT-NIR Spectra Acquisition

Triploid watermelon seed has a hard and thick seed coat, between 0.27 and 0.3 mm depending on seed variety. To ensure that during spectral acquisition, the spectral information of embryo is being accumulated as well, one simple experiment was done. Regarding this issue, from each variety of triploid watermelon seed, 5 seeds were sliced by a surgical knife to obtain the spectral information of whole seed, seed coat, and the embryo. Afterwards, preprocessing methods were applied to find out the spectral peak difference from the mean spectra. 

The spectra of 1800 samples (from each variety 600 seeds) were collected using an FT-NIR spectrometer (Antaris II FT-NIR analyzer, Thermo Scientific Co., Waltham, MA, USA) equipped with an InGaAs detector. A single seed was placed on a customized sample holder which has a hole in the center designed for small solid samples. To reduce the outside light effect, this holder was covered with a black surface. The absorbance spectra of each seed was collected in the wavelength range between 4000 to 10,000 cm^−1^ (1000–2500 nm) at the interval of 4 cm^−1^ spectral resolution. The average spectrum of 32 successive scans of each individual seed was obtained for further analysis.

### 2.3. Between Paper Germination Test

This is an effective germination test, as in the visualization it is easy to count the sprouted seed: how many are just beginning to sprout and how many show no action at all. Due to being naturally aged seed, there were a large number of seeds which were only beginning to sprout and had not reached to the surface properly. The germination rate was 87% for V1, 89% for V2, and 84% for V3. The germination test was conducted following the guidelines of International Seed Testing Association rules [21]. Seeds were placed very carefully depending on seed number from the 96-well plate on a moistened paper towel to determine seed viability after spectral acquisition. All the rolled towels were kept upright in a deep bottom plastic tray and 3 cm of the rolls were covered from the bottom with distilled water and moistened when necessary. The rolled paper towels were stored in the germination cabinet at 25 °C for 14 days. Seeds with a primary root length of 5 mm was considered as viable.

Data from the germination test were recorded into two groups: viable seed and nonviable seed. After 14 days, the seeds which did not show any signs of germination were recorded in the group of non-viable or dormant seeds. However, the main focus of this work was to find out the feasibility of using FT-NIR spectroscopic technique and to make a general model to classify naturally aged viable and non-viable triploid watermelon seed varieties.

### 2.4. Data Preprocessing and Multivariate Analysis

Due to being comprised of broad and overlapping peaks composed of unexpected scattering noise, the raw spectra which was directly captured form FT-NIR spectroscopy was not used directly for multivariate analysis. FT-NIR spectra are highly correlated with neighboring noise caused by light scattering, instrumental drift, and baseline shifts along with slope variation [22]. To remove these aspects, preprocessing methods were applied to smooth the spectra. In the final classification model, the shorter and longer wavelength regions were removed due to containing less information and noisy appearance. Afterwards, the analysis of chemometrics with the combination of partial least discriminant analysis (PLS-DA) was performed by the MATLAB (2016a, The MathWorks, Natrick, MA, USA) software. The multivariate analytical model of partial least square discriminant analysis (PLS-DA) was developed to classify the different varieties of naturally aged triploid watermelon seed according their viability. PLS is used as classification model routinely in applied statistics and performs well [23].

However, there are various preprocessing methods. Three types of normalization (maximum, mean, and range), smoothing (Savitzsky-Golay 1st and 2nd derivatives), multiple scattering correction (MSC), and standard normal variate (SNV) were applied where the original values are transformed and the general assumption of the data set will hold. In this study, to verify the final model to obtain realistic estimation of the prediction performance, a total new data set was used. 

PLS-DA, commonly used for model classification, is the modification form of PLS-R (partial least square regression). It is expressed as:

(1) Y=X×b+E

where, X is an n×p matrix that holds the spectra values of each class, b is the regression coefficient, and E is the error term. In this study, for the construction of PLS-DA model, spectral data of viable and non-viable seeds were arranged in a matrix X while the Y matrix contained an artificial value expressing class as given below:

$$Y=\{\begin{array}{c} 0=sample\;belongs\;to\;nonviable\;group\\ 1=sample\;belongs\;to\;viable\;group \end{array}$$

In order to correctly classify the samples, a baseline was selected as ±0.5 in respect to each group. Samples within the range of ±0.5 from any group were considered as classified in that group. 

To build a linear relationship between the predictors and response variables, both X and Y values are changed by latent variables (LVs).

(2) $$X=TP^{T}+E_{X}$$

(3) $$Y=UQ^{T}+E_{Y}$$

Here, P and Q are loading matrix and T and U are score matrix. $E_{X}$ and $E_{Y}$ are residual matrix of X and Y respectively.

### 2.5. Optimal Variable Selection

The variable selection method was applied to obtain optimal number of variables that would also be used to develop the viability classification model for naturally aged triploid watermelon seed. The main objective of the optimal wavelength selection is to select those wavebands that are composed of meaningful data and eradicate the undesirable wavebands from the spectral data. This will make the technology convenient for industrialized uses with rapid measurements. VIP (variables importance in projection) summarize the X-variables to latent structure in multivariate models. The VIP for the j-th variable is defined as:

$$VIP_{j}=\sqrt{\frac{p\sum_{a=1}^{A}W_{ja}^{2}\times SSY_{a}}{SSY_{total}\times A}}$$

Here, *p* is the number of the variable, $w_{ja}$ is the weight value for the j-th variable of component a, $SSY_{a}$ is the sum of squares of the explained variance for the a-th component, $SSY_{total}$ is the total sum of square explained for dependent variable, and A is the total number of components. The covariance between the dependent and independent variables described by the weight value of the PLS-DA model.

## 3. Result and Discussion

### 3.1. Embryo Information Acquisition

**Figure 2:**
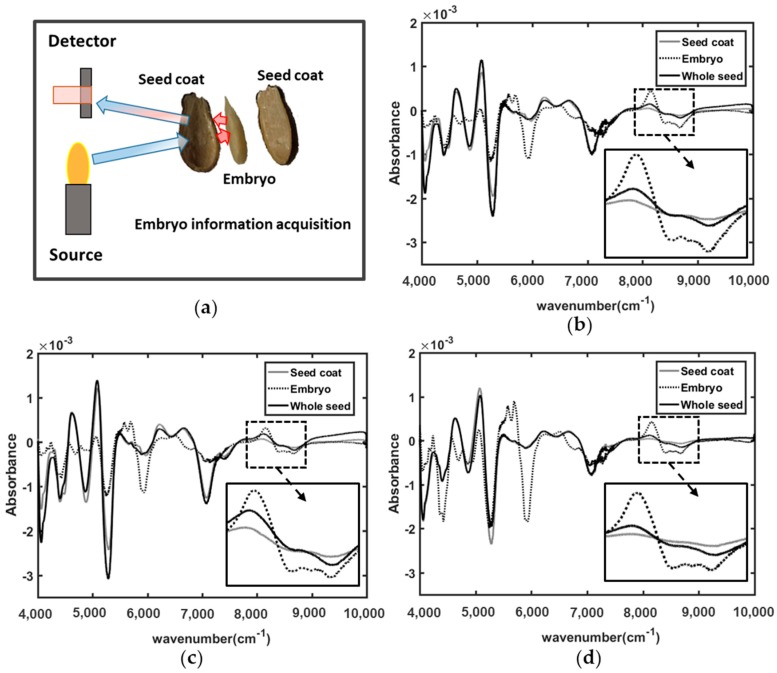
(**a**) Embryo chemical information acquisition in FT-NIR spectroscopic technique; (**b**) V1; (**c**) V2; and (**d**) V3 variety.

Figure 2a illustrated the approach of the spectral acquisition of embryo using FT-NIR spectroscopy. For the three different varieties of triploid watermelon seed, the embryo part has given spectral peak information in similar waveband regions. The waveband region from 4000 to 10,000 cm^−1^ NIR reflectance spectra has shown several spectral absorption peaks of information for the embryos. The seed coat and whole seed spectral information are very similar for all three varieties but slightly different for the embryo absorption spectra. 

From this figure it is clearly demonstrated that, for all the three varieties of seed sample, wavenumber between 8000 and 9000 cm^−1^ have less spectral peak for seed coat whereas embryo has informative spectral peaks. The spectrum of whole seed has shown absorption information between seed coat and embryo with similar pattern of embryo. Hence, FT-NIR spectroscopy is able to provide the information of embryo condition of triploid watermelon seed varieties. Savitzsky-Golay’s first smoothing derivative was applied to visualize the difference clearly. From the Figure 2b–d it can be seen that the NIR spectra throughout the whole range can collect the physiochemical information from the embryo despite the thick seed coat. Moreover, all three varieties provide embryonic information in similar wavenumber region, it would be a great advantage for the general model to detect viable and nonviable seeds. Previous researches proved that, S-G preprocessed spectra always gives more accurate spectral information [24].

### 3.2. Spectral Interpretation

The spectra of 1800 seeds (600 seeds from 3 varieties) were taken by single sample basis and then stored carefully by its number in a 96-well plate. This seed number is followed during germination test also and kept record of germination test as viable or nonviable seed carefully. Until the germination test was performed, seeds were stored in refrigerator at 4 °C, wrapped with paper in a Ziploc bag. 

**Figure 3:**
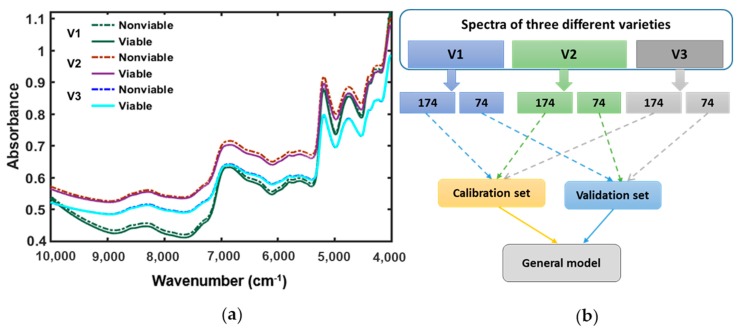
(**a**) FT-NIR spectra collected form three varieties of triploid watermelon seed; (**b**) General model development and validation.

Among the major parts of a dicot seed; seed coat (or testa), and embryo; embryo contains higher protein expression level of globulin proved by previous published researches [25,26,27]. The identification of molecular structure and certain absorption bands related to the functional group are the main important ant aspects of spectroscopy. The raw spectra were collected from naturally aged watermelon seed varieties for the full NIR region between 4000 and 10,000 cm^−1^. Further analysis of this study is done with the application of various preprocessing methods to get high accuracy in model developing due to classifying the naturally aged triploid watermelon seed varieties. The original mean spectra (Figure 3a) of viable and nonviable seed depending on viability tests for three different varieties of triploid watermelon seed were plotted. Figure 3a shows NIR absorption bands for all the varieties up to 4000–7000 cm^−1^, centered at approximately 4536, 4987, 5174, 5376, and 6929 cm^−1^. The absorption band from 4536 to 4981 cm^−1^ assigned for the second overtone of C—H functional group, also has a small bump in the vicinity at 4744 cm^−1^. This absorption band is the characterization of the second overtone of C—H stretching vibration of various functional groups of –CH2, -CH2, -CH=CH- [28]. The similar functional C—H groups are also found in between 5174 and 5376 cm^−1^ NIR reflectance regions. In the absorption band between 6035 and 7458 cm^−1^, there is a spread bump centered at 6929 cm^−1^. This region shows the first overtone of C—H stretching vibration of methyl and methylene group. A series of research studies on this region of NIR spectra proved the correlation with protein [28]. 

In Figure 3a, for each variety of triploid watermelon seed, the viable group has shown lower absorbance than the nonviable group which shows consistency with the result of artificially aged watermelon seed viability detection [15]. Therefore, we can assume that differences in light scattering were observed between naturally aged viable and nonviable triploid watermelon seed varieties. Hence, from the original mean spectra (Figure 3a), it is clear that there is no spectral peak information between the 9000–10,000 cm^−1^ waveband. For further analysis, this range of waveband was omitted. 

Eighteen hundred spectra from the triploid watermelon seed varieties were collected and utilized to make a general model (Figure 3b). Finally, this general model was used for predicting seed viability among these three varieties to investigate the model performance. In the mean spectra for viable and nonviable groups, very minimal spectral difference was observed. Though it did not appear in the spectral depiction, the PLS-DA model considered everything and could detect viable and nonviable seeds at a satisfactory rate for naturally aged triploid watermelon seed. From each of the seed varieties, 248 seed samples (124 viable and 124 nonviable) samples were taken. For model validation 35% of seed samples (74 samples: 37 viable and 37 nonviable) were used, whereas the remaining 65% of data was used as a calibrating model (174 samples: 87 viable and 87 nonviable). Besides, the samples which were used in model calibration, were not used in the validation set.

To make the ‘general model’, a total of 744 samples (372 viable and 372 nonviable) were used from three varieties. As a result, in model calibration, a total of 520 samples (260 viable and 260 nonviable) and 224 samples in the validation set (112 viable and 112 nonviable seed) were used. For the performance test of the “general model”, new seed sample was used as test sample.

### 3.3. PLS-DA Model

To establish the classification model, partial least square discriminant analysis (PLS-DA) was performed in this study, which is suitable when the matrix of predictors has more variables than observations and there is a high correlation among the original predictors [29]. Next, the Savitzsky-Golay 1st smoothing derivative was applied, which performs a local polynomial regression on a series of value to determine the smoothed value. 

**Figure 4:**
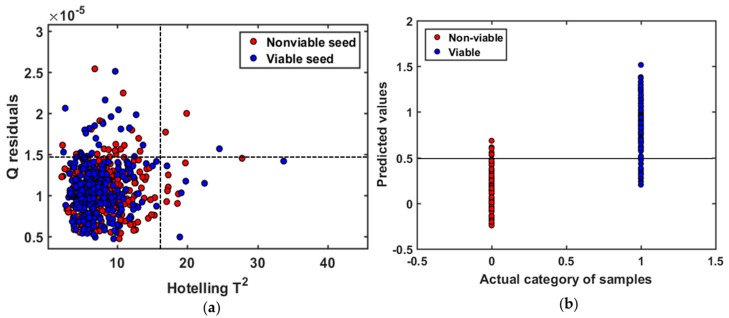
(**a**) Plot of Q residuals versus Hoteling’s T2. Nonviable seed samples are colored in ‘red’ and viable samples in ‘blue’ for the general model; (**b**) classification result of the validation set with Savitzsky-Golay 1st derivative preprocessing at 0.5 threshold value.

For establishing the general model of triploid watermelon seed, Hoteling’s T^2^ ellipse was used to determine the outliers. The Q statistic evaluates that how each sample conforms the model. On the other hand, Hoteling’s T^2^ measures the variation in each sample within the model. In Figure 4a, the plot of Hoteling’s T^2^ versus Q residuals is shown for the general model made from three different varieties of triploid watermelon seed. No samples were found with high residual and high Hoteling’s T^2^ which can be detected as outliers, probably because of customized sample holder. For the first two principle components of each group (viable and nonviable), samples were inside the ellipse with the confidence level of 98.9%. 

The classification parameters of viable and nonviable seed for this general model were collected from PLS-DA model after applying several preprocessing methods. Among them, Savitzsky-Golay 1st derivative provided the highest accuracy, as presented in Table 1. The regression coefficient curve clearly demonstrated that FT-NIR technique contains informative result to differentiate naturally aged viable and nonviable triploid watermelon seed. The PLS-DA model yielded satisfactory accuracies after the application of preprocessing methods, which means there was a difference between naturally aged viable and nonviable triploid watermelon seed varieties. For the general model, the Savitzsky-Golay 1st derivative with a 4-point window exhibits higher model accuracy in validation set (90%). The Savitzsky-Golay derivatives correct the baseline effect in the spectra to remove non-chemical effects. Probably the biggest variation among the baseline of spectra from three different varieties were apparently corrected, thus leading to the higher classification accuracy. The latent variables (LVs) were calculated for the prediction set in terms of lower error rate, where the error rate is the most-common parameter to measure the classification model quality [30]. The classification details of naturally aged triploid watermelon seed, plotted in Figure 4b, demonstrate that viable seed can be distinguished with high accuracy. 

**Figure 5:**
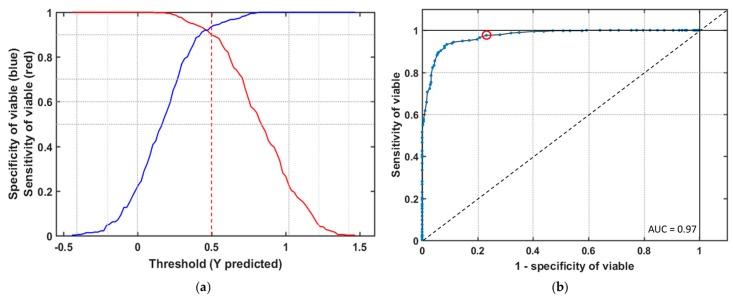
(**a**) Effective threshold selection for partial least discriminant analysis (PLS-DA) based seed viability classification (the line in red color is the sensitivity and blue color is the specificity of viable seed); (**b**) Receiver operating characteristics curve for viable seed detection from the general model.

In this study, this classification model would be used to screen new seed samples (viable and nonviable seed) of triploid watermelon seed. To be confirmed that the classification performance is not influenced by samples, ROC (Receiver Operating Characteristics) curves were adopted to evaluate the classification capability for viable seeds of the model. Figure 5a shows the sensitivity/specificity versus probability cut off point. They cross each other fairly close to the upper horizontal axis, which means the threshold attained from the PLS-DA model has a good sensitivity without losing much specificity. It plots the proportion of true positives against the false positive of all values of the threshold parameter. For a perfect classification method, yield point will present on the upper left corner of the ROC space representing maximum sensitivity and specificity for a group. For random classification, points will appear from the bottom left to top right corner along a diagonal line. Figure 5b shows the ROC curve from the general model made of different varieties of naturally aged viable watermelon seed. As expected from the results of Table 1, the ROC curve for the identification of viable seed, after applying the preprocessing method, has made a satisfactory classification in the detection of a viable group. 

**Figure 6:**
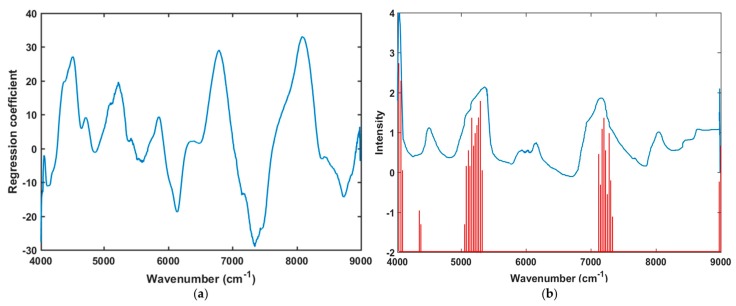
(**a**) Regression coefficient derived from the PLS-DA model; (**b**) The variables selected are represented by the red lines and the average spectrum preprocessed with the Savitzsky-Golay 1st derivative is overlaid to enable comparison of the original data.

The spectral peaks for naturally aged triploid watermelon seed were observed from the regression curve. The regression coefficient was calculated and plotted by the PLS-DA model that interprets different characteristics between naturally aged viable and nonviable triploid watermelon seeds (Figure 6a). In multivariate analysis, the beta coefficient of the PLS model is the most important factor due to informative wavelength selection and the interpretation of results [31]. The highest absolute values of the model with the most important variables is shown by the beta coefficient of the PLS model. In this case, the regression coefficient represents the spectral difference (peaks and valleys) between the viable and non-viable seed groups based on important peaks due to chemical components, and showed high absolute values of the wavelengths that agreed with the mean spectra plot (Figure 3a) and PLS-DA classification result (Table 1). 

Additionally, the important wavenumber region of 4000 to 4194 cm^−1^ reflects for the combination of N–H second overtone stretching vibration and C–H stretch and deformation. Absorption in this region reflects mainly protein moieties like aromatic amino acid (ArNH2) and NH2 [32]. In addition, the varieties show strong spectral peaks in between 5203 to 6400 cm^−1^ which also corresponds to the C–H combination and first overtone of N–H stretching vibration due to absorption by CH2 and protein moieties [28]. Therefore, in our study, protein moieties may be a possible reason for the viability detection of naturally aged triploid watermelon seed. The waveband region between 7403 and 8201 cm^−1^ shows absorption bands for all the varieties of triploid watermelon seed represent the C = O stretch second overtone combined with O–H stretch and HOH deformation, as well a second overtone of O–H bend. Notably, protein and water content show the absorption characteristics in this waveband region [28]. Previous research has revealed that the absorption band in this region correlates more to water than other compounds as viable seeds often retain more bound water [13]. 

According to Shaban (2013) the quality of stored proteins in the seed has a great impact on viability [33]. Various published studies confirmed that there is a direct relationship between seed viability and the amount of seed protein [34,35]. They found a change in stored protein for non-germinated seeds of white lupin seed. A similar result observed for aged bean seeds [36]. The researchers observed gradual disappearance of polypeptide band in bean seed protein profile along with the loss of seed viability. According to other published research, these modifications in protein degradation occurred during seed storage [37]. Furthermore, degradation of seed protein creates a disturbance to the supply of amino acids, which is necessary for the synthesis of new proteins during germination and seedling formation [35]. These statements support the hypothesis that seed viability strongly correlates with the stored protein in naturally aging seeds. Seed moisture content is also related with seed viability. Seeds with critical moisture level lose viability at a faster rate [38].

The variable importance projection (VIP) helps to find out the contribution of each variable in the spectra and describes the relationship between the spectral matrix (X) and response vector (Y) [39]. VIP interpretation contributes to discriminate between naturally aged triploid watermelon seed viability detection from this generalized model. Generally, the performance of the PLS-VIP model depends on the cut-off value to select the relevant predictors. In this study, to minimize the number of variables as much as possible and to develop a fast model with greater accuracy, VIP was applied. Values ranging from 0.8 to 1.5 were examined to select the threshold value, and the best combination of variable number with classification accuracy was achieved at 0.9. The performance of the PLS-DA-VIP model for the calibration and validation set was similar as the model was developed to contain all variables. In Figure 6b, evident peaks were found at 4079, 5261, and 7416 cm^−1^. Hence, the VIP score plot also provided similar information for the chemical change in naturally aged viable and nonviable triploid watermelon seed varieties.

### 3.4. Model Validation with New Data Set

The main interest of this research was not only to make a model, but also to apply the model on a test (new) data set to identify naturally aged viable and nonviable seed. In real-life scenarios, seed suppliers and companies need a general model that can identify viability even though the natural aging of seed. For quality seed marketing, the seed companies need to check seed viability for every single seed before handover to the consumer. For this reason, the three varieties of triploid watermelon seed samples were mixed and 100 random seeds were taken. 

**Figure 7:**
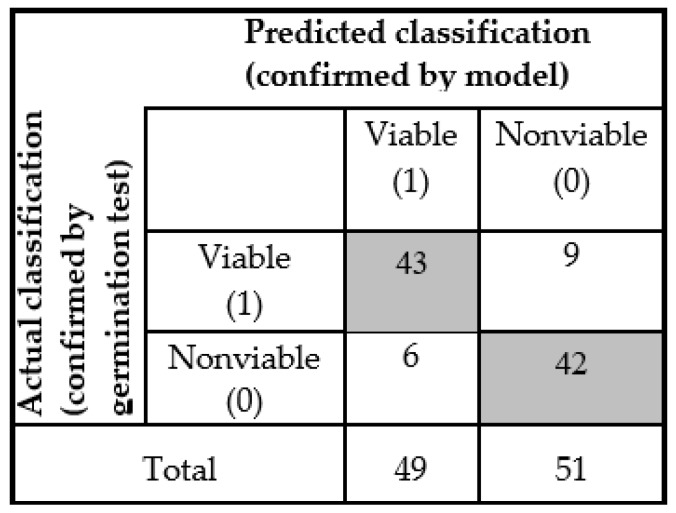
Confusion matrix of test data set measured from general model.

Afterwards, the model was applied on the seed bunch and detected as two groups (viable and nonviable) seeds. These two groups of seeds were again set in the germination cabinet to check the seed viability and match it with the model-based classification result. For the detection of a new seed sample, accuracy was lower than the PLS-DA model, but for the viable seed, all the seedling growth during germination was normal and healthy, which means each seedling will be able to produce mature plant. Here also, we considered 5 mm primary root length for a seed as viable. The number of healthy viable seeds (during germination test) was less in the ‘nonviable’ group from the model detection. The test seed sample detection result was shown by the ‘confusion matrix’ plotted in Figure 7.

The classification parameters were given for the generalized model in Table 2. The new data set sample detection results indicate higher sensitivity and specificity equal to 0.877 and 0.82 respectively. It means that 87.7% of samples (43 out of 49 seed) were correctly classified as viable, and 82% of samples (42 out of 51 seeds) were correctly classified as nonviable seed. The non-error rate (NER) describes the model accuracy, which is 0.85, which means we can accept this classification on the basis of its final aim. The best precision or positive prediction value (PREC) is 1.0 and the false positive rate (FPR) value is 0. Depending on the sensitivity value, the FT-NIR spectroscopic technique gave a promising result to use this generalized model for further analysis to detect naturally aged viable seed.

## 4. Conclusions

Seed viability is one of the most important attributes needed to make a reasonable prediction of seed performance in the field. Therefore, in order to increase the crop yield and thus more production, seed companies and farmers’ organizations are in need of a rapid and non-destructive technique to confirm the seed viability. Various Spectroscopic techniques with chemometric methods have been utilized to discriminate viable seeds from artificially aged nonviable seeds. As discussed previously, there is some physiochemical difference between naturally and artificially aged seeds, which can lead to result misrepresentation and misinterpretation. 

In this work, naturally aged watermelon seeds of three different varieties were used to develop a spectroscopy and multivariate data analysis-based general model to predict seed viability. The ability of the general model was further tested with an external data set collect information at different times, thus to ensure that the model can be used for any variety of watermelon seed (from three used varieties) in the future using the FT-NIR spectroscopic technique in the 4079, 5261, and 7416 cm^−1^ waveband region. However, previous work on the detection of corn and soybean seed viability was not successful using NIRS [10]. It may happen due to the detection limitation of NIRS or seed heterogeneity. Published articles provided the information on viability affected by the degradation of protein and moisture content of seed. Hence the current result provided a good separation of viable seeds from nonviable, and conveys similar information. Since FT-NIR spectroscopy used in this study is a single seed based technique, it is not feasible for viability testing of a bulk amount of seeds. Therefore, based on the results of this feasibility study, an on-line hyperspectral imaging system in the NIR range for rapid viability analysis of bulk seed samples is under production, and upon the successful completion, will ultimately benefit the seed companies as well as farmers.

## Figures and Tables

**Table 1:** Seed viability detection in prediction for the general model.

Model	Samples Used for Model Making	Preprocessing	^1^ LVs	Calibration			Validation		
				Viable	Non-Viable	Total	Viable	Non-Viable	Total
General model made of three varieties	744	Raw	13	198/260	182/260	73.1%	85/112	74/112	72.1%
		Savitzky-Golay 1st	8	253/260	250/260	96.7%	103/112	99/112	90.1%

^1^ LVs, number of latent variables.

**Table 2:** Classification parameters obtained from unknown (test) data set after applying the model.

Test Set Variety	Samples Used	NER/Accuracy	ER	Sensitivity	Specificity	PREC	FPR
Random	100	0.85	0.15	0.877	0.82	0.83	0.17

NER, Non Error Rate; ER, Error Rate; PREC, Positive Predictive Value or Precision; FPR, False Positive Rate.
